# Clinical Features and Long-Term Survival of Metastatic Hepatic Neuroendocrine Neoplasms Secondary to Gastroenteropancreatic Site: An Analysis by Applying the Grading Classification

**DOI:** 10.1155/2020/6572398

**Published:** 2020-09-15

**Authors:** Min Yang, Lin Zeng, Sheng-zhong Hou, Neng-wen Ke, Bo-le Tian, Xu-bao Liu, Bo Xiang, Yi Zhang

**Affiliations:** ^1^Department of Pediatric Surgery, West China Hospital of Sichuan University, Chengdu, Sichuan Province, China; ^2^President & Dean's Office, West China Hospital of Sichuan University, Chengdu, Sichuan Province, China; ^3^Department of Pancreatic Surgery, West China Hospital of Sichuan University, Chengdu, Sichuan Province, China

## Abstract

**Method:**

Data of patients who were surgically treated and clinicopathologically diagnosed as (MH)-NENs secondary to (GEP)-NENs at West China Hospital of Sichuan University from January 2006 to December 2018 were retrospectively collected and analyzed by the grading classification for (GEP)-NENs.

**Results:**

We identified 150 patients with (MH)-NENs secondary to (GEP)-NENs, including 10 patients with G1 NETs, 26 with G2 NETs, 33 with G3 NETs, and 81 with G3 NECs. There were significant differences between patients with G1/G2/G3 NETs and those with G3 NECs, such as age at diagnosis (*P*=0.041), synchronous liver lesion (*P*=0.032), incidental diagnosis (*P*=0.014), tumor largest diameter (*P*=0.047), vascular invasion (*P*=0.017), and extrahepatic metastatic disease (*P*=0.029). The estimated 3-year overall survival for patients with G1 NETs, G2 NETs, G3 NETs, and G3 NECs was 100%, 79.4%, 49.5%, and 20.7%, respectively (*P* < 0.001). The survival of G1 NETs or G2 NETs was significantly better than that of G3 NETs (*P*=0.013, *P*=0.037, respectively) and G3 NECs (*P*=0.001, *P* < 0.001; respectively). Patients with G3 NECs present notably worse survival than those with G3 NETs (*P*=0.012), while survival comparison between G1 NETs and G2 NETs was not statistically different (*P*=0.131). The grading classification for (GEP)-NENs was an effective independent predictor of survival for (MH)-NENs secondary to (GEP)-NENs (hazard ratio: 4.234; 95% confidence intervals: 1.984–6.763; *P*=0.003).

**Conclusion:**

Our demonstration revealed that the grading classification for (GEP)-NENs could well stratify (MH)-NENs secondary to (GEP)-NENs into prognostic groups and supported its wide use in clinical practice.

## 1. Introduction

Neuroendocrine neoplasms (NENs) are a group of amine precursor uptake and decarboxylation neoplasms that are highly heterogeneous with distinct pathological features, biological behaviors, and prognosis [1–3]. NENs could occur in various organs or tissues with neuroendocrine cells, which might secrete bioactive amines or peptide hormones, such as insulin, gastrin, prostaglandins, serotonin, etc. and present different clinical manifestations [1–3]. NENs arising from the digestive tract account for the highest rate of incidence of NENs, in which gastroenteropancreatic neuroendocrine neoplasms [(GEP)-NENs] are the most common (about 65%–67%), followed by those from the bronchopulmonary system (about 27%) [3–5]. Studies have indicated the incidence of NENs has increased obviously in the past decades, probably due to the development of endoscopic and radiological imaging techniques, the improvement of clinical awareness, and modified diagnostic techniques for NENs [5–7].

NENs often grow to an advanced stage with either locally unresectable primary tumors or distant metastasis on admission due to the atypical or unobvious clinical manifestations, which are usually difficult to be discovered in early days [5–8]. With the unique anatomical characteristics, the liver is the most common site of metastasis for NENs, especially for (GEP)-NENs. It has been reported that nearly 80% of hepatic neuroendocrine neoplasms [(H)-NENs] are metastatic with synchronous or metachronous masses in primary endocrine sites [6, 9], while primary hepatic neuroendocrine neoplasms [(PH)-NENs] are extremely rare, accounting only for 0.3%–4% of all NENs and 0.28%–0.46% of all hepatic malignancies [7, 10]. Also, metastatic hepatic neuroendocrine neoplasms [(MH)-NENs] may be the first manifestation in up to 40–90% of cases, which decreases the survival of NENs in varying degrees [11, 12]. With the significantly rising incidence of NENs in the past years [5–7], there is still few available data on the clinical features and long-term survival of MH-NENs secondary to (GEP)-NENs.

On the other hand, World Health Organization (WHO) histologically classified (GEP)-NENs into three main groups in 2010 based on the cut-off point of mitotic rate and Ki‐67 proliferative index: G1 neuroendocrine tumors (NETs), G2 NETs, and G3 neuroendocrine carcinoma (“G3 NECs”) [13]. This grading classification for (GEP)-NENs is derived from the one proposed by European Neuroendocrine Tumor Society in 2006 [14], which was recently proven to be a little out-of-date [15–17], although it was widely used thereafter [18–21]. In 2017, based on both morphological differentiation and grading upon proliferation rate, the WHO officially updated its grading system which redefined pancreatic NENs [(P)-NENs] into 2 main categories and 4 groups: well-differentiated tumors (i.e., G1/G2/G3 NETs) and poorly differentiated carcinomas (i.e., G3 NECs) [22]. This new grading system was mainly referred to as (P)-NENs, not those originated from gastrointestinal NENs [(GI)-NENs], although it has still been suggested when analyzing tumors' pathological features [22]. However, whether this grading classification for (P)-NENs or (GI)-NENs is also practical for grouping patients and predicting prognosis of (MH)-NENs secondary to (GEP)-NENs is still unclear.

In this study, based on the relevant data from a large Chinese single institute, we reviewed our experience with (MH)-NENs secondary to (GEP)-NENs, with the primary goal of evaluating the prognostic relevance of the grading classification. To accomplish this, we summarized and compared the clinicopathological features and long-term survival of (MH)-NENs secondary to (GEP)-NENs between each grading group.

## 2. Materials and Methods

### 2.1. Patients Enrollment

Our research was approved by the local ethics committee, and a written informed consent was obtained on admission from all patients in accordance with the general principles of the Helsinki Declaration [23]. Patients who were both clinically and pathologically diagnosed as (MH)-NENs secondary to (GEP)-NENs at West China Hospital of Sichuan University from January 2006 to December 2018 were systematically reviewed from their electronic or paper-based medical records, as we ever did in our previous studies [24, 25]. Patients with a clinical suspicion of (H)-NENs but without a pathological identification were not enrolled in this study. Patients with (MH)-NENs clinically originated from other organs or tissues, such as lung, were also excluded. Patients with (PH)-NENs were excluded as well. For included cases, data of sex, age, clinical presentation, imaging examination, surgical procedures, postoperative outcomes, and so on, were all retrospectively collected and analyzed.

### 2.2. Tumors Feature

The diagnosis of (H)-NENs was performed by surgical specimens or intraoperative biopsy from tumor tissues, which were routinely stained with hematoxylin-eosin and immunohistochemical methods and afterward systematically reviewed by expert pathologists in our institution. The diagnosis of (MH)-NENs secondary to (GEP)-NENs was clinically based on the synchronous or metachronous pathological confirmation of NENs from the gastroenteropancreatic site through the first or second operation. The histopathologic analyzing results (tumor size and location, major vessel invasion, lymph node invasion, portal vein thrombosis, extrahepatic invasion, surgical margin, morphological feature, differentiated degree, immunohistochemical staining examination for mitotic count, Ki-67 positive index, etc.) were all documented. In terms of the tumor grades, (MH)-NENs secondary to (GEP)-NENs in the present study were pathologically classified into G1 NETs, G2 NETs, G3 NETs, and G3 NECs according to the grading classification for (P)-NENs or (GI)-NENs [22].

### 2.3. Statistical Analysis

Follow-up was mainly conducted by telephone, e-mail, or outpatient clinic review between June 2019 and January 2020, leading to a median follow-up time of 38.2 months (range 12.5 months–146.8 months). Overall survival (OS) was calculated as the number of months from the date of surgery (i.e., the date of diagnosis of liver metastatic disease for enrolled patients) to the date of last contact or time of death and presented as either median survival time (MST) or estimated 3-year OS with a hazard ratio (HR) or 95% confidence intervals (CIs). Patients who were lost to follow-up were excluded in the final survival analysis models. Quantitative variables were reported as medians with ranges and compared by Student's *t*-tests, while categorical variables were presented as numbers with frequencies or proportions (%) and compared by Chi-square tests. OS was estimated using Kaplan–Meier (K–M) methods and compared using the log-rank test. Univariate and multivariate analyses were performed by Cox regression proportional hazards model to explore the potential prognosis-related factors for the OS of (MH)-NENs secondary to (GEP)-NENs. Statistical analyses were performed using IBM SPSS version 22.0 software. A two-side *P* value of <0.05 was considered statistically significant.

## 3. Results

### 3.1. Patient Demographics and Clinicopathological Characteristics

According to the inclusion criteria of our present study, we identified 150 consecutive patients who were clinicopathologically diagnosed as (MH)-NENs secondary to (GEP)-NENs at West China Hospital of Sichuan University from January 2006 to December 2018 (Table 1). Our research consisted of 90 male patients and 60 females, with a median age of 56 years. (range 42 years–74 years). Ninety-six (MH)-NENs were diagnosed synchronously with the primary lesions from gastroenteropancreatic site, while the rest of 54 metachronous cases were detected sometime after the first operation of (GEP)-NENs. There were 30, 12, and 44 (MH)-NENs which, respectively, originated from the stomach, small intestine, and large intestine (i.e., (GI)-NENs), while 64 cases were secondary to the pancreas (i.e., (P)-NENs). Eight-four patients with (MH)-NENs present nonspecific symptoms or signs related to hormone overproduction, while 48 ones were detected incidentally by routine physical examination or postoperative review. One hundred and ten patients showed more than 1 visible hepatic lesions, with a median sum of the largest diameter of 13 cm (range 2 cm–24 cm). Multifocal lesions of 92 cases were located in both right and left half liver, while 127 patients present over 50% estimated liver involvement. Fifty-seven and 30 patients were, respectively, treated by resection only and ablation only, while 41 ones were resected simultaneously with ablation during operation. The surgical margin of 57 (MH)-NENs was both grossly and microscopically negative (i.e., R0 resection). Ninety-three and 128 patients, respectively, received pre- and postoperative systemic therapy. There were, respectively, 57, 65, 27, and 42 patients who showed regional lymph node metastases, portal vein tumor thrombus, vascular invasion, and extrahepatic metastatic disease.

### 3.2. Subgroup Analysis according to the Grading Classification

Of 150 assessable patients with (MH)-NENs secondary to (GEP)-NENs, we defined 10 patients with G1 NETs, 26 with G2 NETs, 33 with G3 NETs, and 81 with G3 NECs by the grading classification for (P)-NENs or (GI)-NENs (Table 1). There were distinct characteristics and clinicopathological differences between patients with G1/G2/G3 NETs and those with G3 NECs in our study. Notably, patients with G1/G2/G3 NETs were notably younger than those with G3 NECs (*P*=0.041). G3 NECs present a more synchronous liver lesion in comparison to G1/G2/G3 NETs (*P*=0.032). G3 NECs were detected more incidentally than those with G1/G2/G3 NETs (*P*=0.014). The median sum of the largest diameter of all visible hepatic lesions with G1/G2/G3 NETs was significantly smaller than that of lesions with G3 NECs (*P*=0.047). G3 NECs present more vascular invasion and extrahepatic disease compared with those with G1/G2/G3 NETs (*P*=0.017, *P*=0.029, respectively). It seemed that both G1/G2/G3 NETs and G3 NECs tended to originate from the large intestine and pancreas (69.6%, 74.2%, respectively), with a preferable metastatic location in the right half liver (85.5%, 91.3%; respectively). Other comparisons between G1/G2/G3 NETs and G3 NECs, such as patients' sex, tumor type, multifocal lesions, estimated liver involvement, surgical procedure and margin, pre- and postoperative systemic therapy, regional lymph node metastases, and portal vein tumor thrombus, were not statistically significant (*P* > 0.05).

### 3.3. Survival Analysis for (MH)-NENs Secondary to (GEP)-NENs

**Figure 1:**
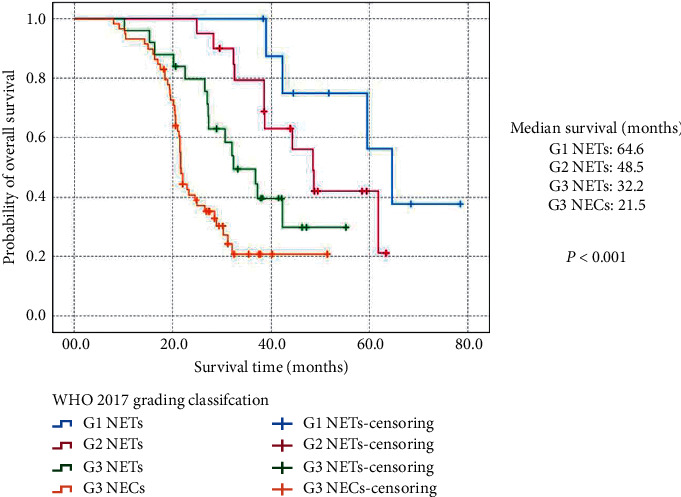
Kaplan–Meier estimates for the overall survival of metastatic hepatic neuroendocrine neoplasms secondary to the gastroenteropancreatic site, according to the grading classification proposed for gastroenteropancreatic neuroendocrine neoplasms.

When the follow-up ended in January 2020 (Table 1), thirty-seven patients were out of contact, including 1 patient with G1 NETs, 6 with G2 NETs, 8 with G3 NETs, and 22 with G3 NECs. Of the 113 patients in touch, there were 72 deaths due to tumor progression or burden, including 4 G1 NETs, 11 G2 NETs, 15 G3 NETs, and 42 G3 NECs. The estimated 3-year OS for the entire cohort with (MH)-NENs secondary to (GEP)-NENs was 45.6%, with a MST of 31.9 months (95% CIs: 24.8 months–39.0 months). The estimated 3-year OS for patients with G1 NETs, G2 NETs, G3 NETs, and G3 NECs was 100%, 79.4%, 49.5%, and 20.7%, with a MST of 64.6 months (95% CIs: 53.8 months–75.4 months), 48.5 months (95% CIs: 40.9 months–56.1 months), 32.2 months (95% CIs: 23.2 months–41.2 months), and 21.5 months (95% CIs: 20.9 months–22.1 months), respectively (*P* < 0.001; Figure 1). Further analysis among each new grading group indicated that survival of G1 NETs or G2 NETs was significantly better than that of G3 NETs (*P*=0.013, *P*=0.037, respectively) and G3 NECs (*P*=0.001, *P* < 0.001; respectively). Patients with G3 NETs present notably longer survival time than those with G3 NECs (*P*=0.012), while survival comparison between G1 NETs and G2 NETs was not statistically different (*P*=0.131).

**Figure 2:**
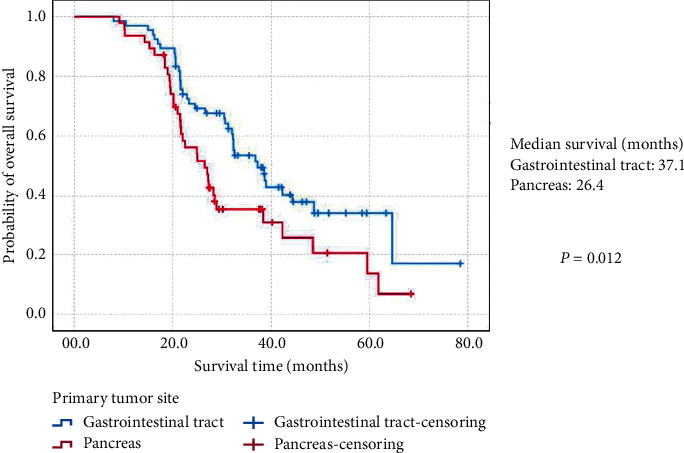
Kaplan–Meier estimates for the overall survival of metastatic hepatic neuroendocrine neoplasms secondary to the gastroenteropancreatic site, according to the primary tumor site.

**Figure 3:**
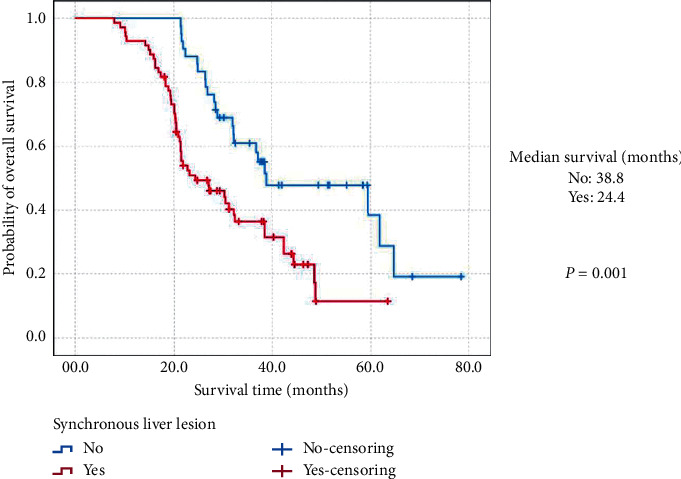
Kaplan–Meier estimates for the overall survival of metastatic hepatic neuroendocrine neoplasms secondary to the gastroenteropancreatic site, according to the synchronous liver lesion.

**Figure 4:**
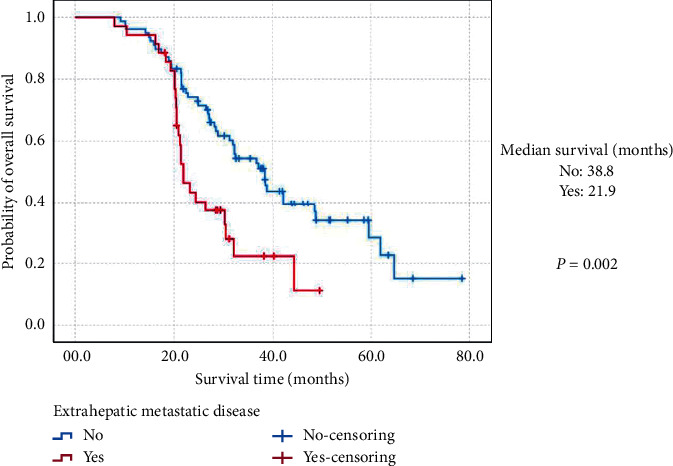
Kaplan–Meier estimates for the overall survival of metastatic hepatic neuroendocrine neoplasms secondary to the gastroenteropancreatic site, according to extrahepatic metastatic disease.

Moreover, the estimated 3-year OS and MST of (MH)-NENs originated from the gastrointestinal tract was 53.6% and 37.1 months (95% CIs: 30.3 months–43.9 months), which was significantly better than 35.0% and 26.4 months (95% CIs: 21.2 months–31.6 months) of those secondary to the pancreas (*P*=0.012; Figure 2). (MH)-NENs diagnosed synchronously with the primary lesions showed a much worse survival than metachronous tumors (36.3% vs. 61.0%; 24.4 months (95% CIs: 15.2 months–33.6 months) vs. 38.8 months (95% CIs: 16.9 months–60.7 months); *P*=0.001; Figure 3). The 3-year OS and MST of (MH)-NENs with extrahepatic metastatic disease was 22.3% and 21.9 months (95% CIs: 19.7 months–24.1 months), compared statistically shorter than those without (54.4%; 38.4 months (95% CIs: 31.9 months–44.8 months); *P*=0.002; Figure 4).

### 3.4. Prognostic Factors of (MH)-NENs Secondary to (GEP)-NENs

According to our demonstration (Table 2), patients' sex (HR: 1.253; 95% CIs: 0.877–3.013; *P*=0.647) and age (HR: 0.992; 95% CIs: 0.436–1.354; *P*=0.518), tumor largest diameter (HR: 1.011; 95% CIs: 0.457–1.504; *P*=0.083), and preoperative systemic therapy (HR: 1.231; 95% CIs: 0.514–1.993; *P*=0.102) were not notably associated with the survival of patients with (MH)-NENs secondary to (GEP)-NENs. Moreover, tumor type (HR: 1.342; 95% CIs: 0.539–2.492; *P*=0.047), incidental diagnosis (HR: 1.645; 95% CIs: 0.636–3.078; *P*=0.015), multifocal lesions (HR: 1.412; 95% CIs: 0.514–2.009; *P*=0.036), estimated liver involvement (HR: 1.541; 95% CIs: 0.654–2.463; *P*=0.043), surgical procedure (HR: 1.443; 95% CIs: 0.623–2.354; *P*=0.043), postoperative systemic therapy (HR: 1.402; 95% CIs: 0.537–2.114; *P*=0.035), regional lymph node metastases (HR: 1.834; 95% CIs: 0.863–2.942; *P*=0.042), portal vein tumor thrombus (HR: 2.034; 95% CIs: 1.112–3.142; *P*=0.039), and vascular invasion (HR: 1.952; 95% CIs: 0.885–3.432; *P*=0.017) were only statistically significant on univariate analysis. On multivariable analysis, synchronous liver lesion (HR: 2.012; 95% CIs: 1.092–3.475; *P*=0.014), pancreatic primary site (HR: 1.882; 95% CIs: 1.003–3.825; *P*=0.031), R1/R2 resection (HR: 1.764; 95% CIs: 0.743–4.111; *P*=0.007), extrahepatic metastatic disease (HR: 3.053; 95% CIs: 1.473–5.082; *P*=0.027), and G3 NECs by the grading classification (HR: 4.234; 95% CIs: 1.984–6.763; *P*=0.003) were each related to worse long-term survival of patients with (MH)-NENs secondary to (GEP)-NENs.

## 4. Discussion

As we mentioned before, (H)-NENs are rather uncommon, in which (MH)-NENs secondary to (GEP)-NENs account for their majority [3, 6, 7, 9, 10]. According to the inclusive criteria, we hereby identified 150 patients with (MH)-NENs secondary to (GEP)-NENs, which had a slight male predominance with a median age of 56 years (range 42 years–74 years.). These demographic characteristics were much close to the reports by previous studies [26, 27]. In our study, as listed in Table 1, 64 (MH)-NENs were secondary to the pancreas (42.7%), 96 cases were diagnosed synchronously with the primary lesions (64%), 127 cases showed over 50% estimated liver involvement (84.7%), and 42 cases simultaneously present extrahepatic invasion at diagnosis (28%). Xiang et al. reported in their research that 44.9% of (MH)-NENs originated from the pancreas, 65.4% of cases present synchronous liver metastasis, 82.5% of cases had over 50% estimated liver involvement, and 11.1% of cases present extrahepatic invasion [27]. The estimated 3-year OS for patients in our study was 45.6%, which was a little worse than what has been reported in previous studies [26, 27], probably due to the stricter inclusion criteria that (MH)-NENs in the present study was just secondary to the gastroenteropancreatic system, rather than other metastatic sites of NENs.

On the other hand, WHO in 2010 divided (GEP)-NENs into G1 NETs, G2 NETs, and “G3 NECs” based on mitotic rate and Ki‐67 proliferative index, whose clinical value has been validated by subsequent studies [13, 18–21]. However, recent studies have focused on the heterogeneity of WHO 2010 “G3 NECs” group, which might consist of morphologically well-differentiated NETs with a high proliferative rate and true poorly differentiated NECs with small-cell or large-cell features for NENs both originating from the pancreas [15, 16, 25, 28] and the gastrointestinal tract [29–33]. In 2017, based on some established research results on histopathologic criteria to better predict the tumor's grade and biological behaviors, WHO redefined (P)-NENs into well-differentiated tumors of G1 NETs, G2 NETs, G3 NETs and poorly differentiated carcinomas of G3 NECs, referring to both morphological differentiation and grading upon proliferation rate, while those changes for (GI)-NENs have also been suggested although they have not been officially introduced [22, 34].

Till now, there is not a specific grading system for (H)-NENs (either (PH)-NENs or (MH)-NENs) due to the rarity of their epidemiology or the heterogeneity of primary tumors. Considering the homogeneity of metastasis lessons and primary tumors, WHO grading classifications originally proposed for (P)-NENs or (GI)-NENs have also been introduced to (MH)-NENs secondary to (GEP)-NENs [13, 14, 22]. Using the WHO 2010 grading classification, Lv et al. reported that the percentage of G1, G2, and G3 among patients with (H)-NENs was, respectively, 4.94%, 25.93%, and 69.13%, with a separate MST of 40.82 months, 51.87 months, and 33.80 months [26]. There was a significant difference in the OS when comparing G1/G2 NETs with “G3 NECs” (*P*=0.011), while the survival difference between G1 NETs and G2 NETs was not statistically significant (*P* > 0.05) [26]. Moreover, when analyzing the clinicopathological features of 112 patients with (MH)-NENs originating from the digestive tract, Jiao et al. retrospectively enrolled 3 patients with WHO 2010 G1 NETs, 18 with G2 NETs, and 91 with “G3 NECs” [35]. Interestingly, Jiao et al. identified 23 cases with G3 NETs in their “G3 NECs” category, which were morphologically well-differentiated with mean Ki‐67 proliferative index of 44% (range 25%–60%) [35]. Jiao et al. also demonstrated that the MST of G3 NETs for patients with (MH)-NENs originating from the digestive tract was 24.0 months, which was notably longer than 8.0 months of G3 NECs group (*P* < 0.01) [35].

The clinical value of the grading classification for (P)-NENs or (GI)-NENs has not been rigorously validated before for patients with (MH)-NENs secondary to (GEP)-NENs. In the present study, by applying the new grading classification to (MH)-NENs secondary to (GEP)-NENs, we distributed all eligible patients into 4 groups for the first time, in which 10 patients with G1 NETs, 26 with G2 NETs, 33 with G3 NETs and 81 with G3 NECs were respectively identified. According to our analysis, significant differences between G1/G2/G3 NETs and G3 NECs were detected (Table 1), such as those of age at diagnosis (*P*=0.041), the synchronous liver lesion (*P*=0.032), incidental diagnosis (*P*=0.014), tumor largest diameter (*P*=0.047), vascular invasion (*P*=0.017), and extrahepatic metastatic disease (*P*=0.029), which have resulted in different survival among each grading group (*P* < 0.001; Figure 1). Especially, the survival of G3 NETs was notably better than those with G3 NECs (*P*=0.012), but much worse than that of G1 and G2 NETs (*P*=0.013, *P*=0.037; respectively), whereas survival of G3 NECs was statistically worse than that of G1/G2/G3 NETs (*P*=0.001, *P* < 0.001, *P*=0.012; respectively). Finally, we demonstrated that synchronous liver lesion, primary tumor site, surgical margin, extrahepatic metastatic disease, and grading classification were effective independent predictors of OS for patients with (MH)-NENs secondary to (GEP)-NENs (Table 2). Our analyses were essentially in agreement with the results by previous studies [27, 35].

Although we evaluated the prognostic relevance of the grading classification for patients with (MH)-NENs secondary to (GEP)-NENs, we still acknowledged the limitations of our study. First of all, we confined MH-NENs to those only secondary to (GEP)-NENs, which will be of uncertain value for MH-NENs originating from other metastasis sites of NENs, such as lung, adrenal gland, etc. Then, the study population was relatively small, especially those with G1 or G2 NETs, which may affect the accuracy of their survival analysis. Thirdly, because the time span of our study was too long, the detailed data of each patient's medical therapy was rather difficult to be collected, whose clinical effect would not be reflected accurately. Finally, the retrospective nature of the study prevented us from obtaining some detailed information, such as tumor recurrence, which still needs to be further discussed.

## 5. Conclusion

In conclusion, by applying the grading classification for (P)-NENs or (GI)-NENs, we analyzed the clinical features and long-term survival of patients with (MH)-NENs secondary to (GEP)-NENs. We found that G3 NECs behaved more aggressively than G1/G2/G3 NETs. Furthermore, we confirmed that synchronous liver lesion, pancreatic primary site, R1/R2 resection, extrahepatic metastatic disease, and G3 NECs category were significantly associated with worse OS of patients with (MH)-NENs secondary to (GEP)-NENs. In a word, our demonstration indicated that the grading classification for (P)-NENs or (GI)-NENs could well stratify (MH)-NENs secondary to (GEP)-NENs into prognostic groups and supported its wide use in clinical practice.

## Figures and Tables

**Table 1:** Clinical features of (MH)-NENs secondary to (GEP)-NENs according to the grading classification for (P)-NENs or (GI)-NENs^A^.

Characteristics	NET G1 (*N* = 10)	NET G2 (*N* = 26)	NET G3 (*N* = 33)	NEC G3 (*N* = 81)	All (*N* = 150)	*P* value^B^
Sex, male, *n* (%)	6 (60.0%)	15 (57.7%)	19 (57.6%)	50 (61.7%)	90 (60.0%)	0.154
Age at diagnosis, years						
Median (range)	49 (42–59)	53 (44–64)	55 (42–69)	59 (45–74)	56 (42–74)	0.041
Synchronous liver lesion	5 (50.0%)	15 (57.7%)	20 (60.6%)	56 (69.1%)	96 (64.0%)	0.032
Primary tumor site						
Stomach	2 (20.0%)	5 (19.2%)	6 (18.2%)	17 (20.9%)	30 (20.0%)	0.166
Small intestine	1 (10.0%)	3 (11.5%)	4 (12.1%)	4 (4.9%)	12 (8.0%)	
Large intestine	3 (30.0%)	8 (30.8%)	8 (24.2%)	25 (30.8%)	44 (29.3%)	
Pancreas	4 (40.0%)	10 (38.5%)	15 (45.5%)	35 (43.4%)	64 (42.7%)	
Nonfunctional tumor	6 (60.0%)	13 (50.0%)	18 (54.5%)	47 (58.0%)	84 (56.0%)	0.209
Incidental diagnosis	3 (30.0%)	6 (23.1%)	10 (30.3%)	29 (35.8%)	48 (32.0%)	0.014
Multifocal lesions^C^	8 (80.0%)	17 (65.4%)	25 (75.7%)	60 (74.1%)	110 (73.3%)	0.512
Tumor diameter^D^						
Median (range)	8 (2–9)	9 (2–15)	11 (3–17)	15 (4–24)	13 (2–24)	0.047
Tumor location						
Left half liver	1 (10.0%)	4 (15.4%)	5 (15.1%)	7 (8.7%)	17 (11.3%)	0.634
Right half liver	3 (30.0%)	6 (23.1%)	9 (27.3%)	23 (28.4%)	41 (27.4%)	
Both	6 (60.0%)	16 (61.5%)	19 (57.6%)	51 (62.9%)	92 (61.3%)	
Estimated liver involvement						
≤50%	3 (30.0%)	7 (26.9%)	8 (24.2%)	20 (24.7%)	40 (26.7%)	0.591
Surgical procedure						
Resection	3 (30.0%)	10 (38.5%)	12 (36.4%)	32 (39.5%)	57 (38.0%)	0.093
Ablation	3 (30.0%)	6 (23.1%)	7 (21.1%)	14 (17.3%)	30 (20.0%)	
Both	2 (20.0%)	6 (23.1%)	9 (27.3%)	24 (29.6%)	41 (27.3%)	
Others^E^	2 (20.0%)	4 (15.3%)	5 (15.2%)	11 (13.6%)	22 (14.7%)	
Surgical margin						
R0^F^	4 (40.0%)	11 (42.3%)	12 (36.4%)	30 (37.1%)	57 (38.0%)	0.135
Preoperative systemic therapy	6 (60.0%)	16 (61.6%)	22 (66.7%)	49 (60.5%)	93 (62.0%)	0.542
Postoperative systemic therapy	8 (80.0%)	20 (77.0%)	28 (84.8%)	72 (88.9%)	128 (85.3%)	0.436
Regional lymph node metastases	3 (30.0%)	10 (38.5%)	12 (36.4%)	32 (39.5%)	57 (38.0%)	0.401
Portal vein tumor thrombus	4 (40.0%)	12 (46.2%)	14 (42.4%)	35 (43.2%)	65 (43.3%)	0.803
Vascular invasion	1 (10.0%)	3 (11.5%)	5 (15.2%)	18 (22.2%)	27 (18.8%)	0.017
Extrahepatic metastatic disease	2 (20.0%)	5 (19.2%)	8 (24.2%)	27 (33.3%)	42 (28.0%)	0.029
Out of contact	1 (10.0%)	6 (23.1%)	8 (24.2%)	22 (27.2%)	37 (24.7%)	NA
Dead at follow-up	4 (44.4%)	11 (55.0%)	15 (60.0%)	42 (71.2%)	72 (63.7%)	NA
Estimated 3-year OS	100%	79.4%	49.5%	20.7%	45.6%	<0.001
MST, months	64.6	48.5	32.2	21.5	31.9	<0.001

^A^These characteristics of (MH)-NENs secondary to (GEP)-NENs were mainly based on the preoperative imaging examinations, intraoperative surgical findings, and postoperative pathological analysis. ^B^Referring to the comparison between those with NET G1/G2/G3 and those with NEC G3 wherever possible. ^C^Referring to no less than 1 metastatic lesion in the liver. ^D^Referring to the sum of the largest diameter of all visible hepatic lesions. ^E^Referring to some uncommon procedures, such as alcohol injection and liver transplantation, etc. ^F^Referring to radical resections with both grossly and microscopically negative surgical margins. (MH)-NENs: metastatic hepatic neuroendocrine neoplasms; (GEP)-NENs: Gastroenteropancreatic neuroendocrine neoplasms; WHO: World Health Organization; NET: neuroendocrine tumor; NEC: neuroendocrine carcinoma; ^G^grading; TNM: tumor-node-metastasis; AJCC: American Joint Committee On Cancer; ^NA^not applicable; OS: overall survival; MST: median survival time.

**Table 2:** Univariate and multivariate analysis of factors with the OS of (MH)-NENs secondary to (GEP)-NENs using Cox Regression proportional hazard models.

Variable	Univariate analysis		Multivariate analysis	
	HR (95% CIs)	*P* value	HR (95% CIs)	*P* value
Sex				
Male^A^				
Female	1.253 (0.877–3.013)	0.647		
Age at diagnosis				
<Median				
≥Median	0.992 (0.436–1.354)	0.518		
Synchronous liver lesion				
No				
Yes	1.874(0.752–3.984)	0.008	2.012 (1.092–3.475)	0.014
Primary tumor site				
Gastrointestinal tract				
Pancreas	1.553 (0.689–3.457)	0.015	1.882 (1.003–3.825)	0.031
Tumor type				
Functional				
Nonfunctional	1.342 (0.539–2.492)	0.047	1.115 (0.445–2.834)	0.106
Incidental diagnosis				
Yes				
No	1.645 (0.636–3.078)	0.015	1.368 (0.557–2.843)	0.085
Multifocal lesions				
No				
Yes	1.412 (0.514–2.009)	0.036	1.212 (0.413–1.852)	0.098
Estimated liver involvement (*N* = 108)				
≤50%				
>50%	1.541 (0.654–2.463)	0.043	1.124 (0.643–2.547)	0.674
Tumor largest diameter				
<Median				
≥Median	1.011 (0.457–1.504)	0.083		
Surgical procedure				
Resection				
Ablation	1.443 (0.623–2.354)	0.043	0.942 (0.325–2.012)	0.114
Surgical margin				
R0				
R1/R2	2.135 (0.994–4.413)	<0.001	1.764 (0.743–4.111)	0.007
Preoperative systemic therapy				
Yes				
No	1.231 (0.514–1.993)	0.102		
Postoperative systemic therapy				
Yes				
No	1.402 (0.537–2.114)	0.035	1.132 (0.446–1.764)	0.236
Regional lymph node metastases				
No				
Yes	1.834 (0.863–2.942)	0.042	1.435 (0.567–2.715)	0.157
Portal vein tumor thrombus				
No				
Yes	2.034 (1.112–3.142)	0.039	1.573 (0.894–2.064)	0.159
Vascular invasion				
No				
Yes	1.952 (0.885–3.432)	0.017	1.562 (0.645–2.084)	0.103
Extrahepatic metastatic disease				
No				
Yes	2.143 (1.249–3.985)	0.002	3.053 (1.473–5.082)	0.027
Tumor grade by the grading classification				
NET G1/G2/G3			ss	
NEC G3	2.653 (1.419–4.255)	<0.001	4.234 (1.984–6.763)	0.003

^A^The above one of the related variable was regarded as a reference in Cox analysis. OS: overall survival; (MH)-NENs: metastatic hepatic neuroendocrine neoplasms; (GEP)-NENs: Gastroenteropancreatic neuroendocrine neoplasms; HR: hazard ratio; CIs: confidence intervals; NET: neuroendocrine tumors; NEC: neuroendocrine carcinoma; G: grading.

## Data Availability

The data of our present research could not be shared at this time as the data also form part of an ongoing study, while they could be available in the near future from the corresponding author upon request after the accomplishment of our ongoing study.
